# MutexaGPT: An Intuition-to-Design Translator for Physics-based Enzyme Engineering

**DOI:** 10.21203/rs.3.rs-10072540/v1

**Published:** 2026-06-19

**Authors:** Qianzhen Shao, Yinjie Zhong, Sebastian Stull, Xinchun Ran, Ning Ding, Kieran Nehil-Puleo, Ruizhe Yao, Han Xu, Zhongyue J. Yang

**Affiliations:** 1 Department of Chemistry, Vanderbilt University, Nashville, Tennessee 37235, United States; 2 Center for Structural Biology, Vanderbilt University, Nashville, Tennessee 37235, United States; 3 Vanderbilt Institute of Chemical Biology, Vanderbilt University, Nashville, Tennessee 37235, United States; 4 Data Science Institute, Vanderbilt University, Nashville, Tennessee 37235, United States; 5 Department of Chemical and Biomolecular Engineering, Vanderbilt University, Nashville, Tennessee 37235, United States; 6 Department of Computer Science, Vanderbilt University, Nashville, Tennessee 37235, United States; 7 Interdisciplinary Materials Science Program, Vanderbilt University, Nashville, Tennessee 37235, United States

**Keywords:** enzyme engineering, EnzyHTP, large-language model, LLM agent

## Abstract

Physical intuitions about how enzyme structure and dynamics influence its function and property have enabled successful engineering outcomes, yet a systematic approach remains unknown for translating these qualitative and abstract “thoughts” into quantitative, actionable principles that lead to designs. Here we introduce MutexaGPT, an open-access, multi-agent large language model (LLM) platform that translates enzyme engineering intuition to physics-based simulations and thus variant designs. Through a web interface, MutexaGPT takes users’ intuition-driven requests (expressed in plain English) as input, and then leverages its LLM agents (i.e., QuestionAnalyzer, WorkPlanningBoard, and ResultExplainer) to comprehend the request and elicit missing information, construct physics-based models, configure and execute high-throughput molecular modeling workflows, and eventually convert the molecular modeling results into actionable design proposals (such as a smart mutation library). An automated evaluation framework was established to systematically benchmark the prompt engineering strategies that allow each individual agent to achieve an optimal performance. We further demonstrate the utility of MutexaGPT in two protein engineering tasks. For the task of engineering halide methyltransferase towards bulkier substrates, MutexaGPT converted a cavity-engineering intuition into a smart library design that shows a 40% hit rate and around 4-fold activity improvement over a baseline strategy. For the task of engineering bidomain amylase for enhanced activity at lower temperature, MutexaGPT translated a statistics-based intuition into cold-adapted amylase variants that show 1.7-fold and 3.7-fold activity enhancement at 0 °C (experimentally validated). These results establish MutexaGPT as an intuition-to-design translator that integrates human creativity with high-throughput molecular modeling to democratize physics-guided, intuition-driven enzyme engineering.

## 1. Introduction

Physical intuition^1, 2^ has long been a cornerstone of enzyme engineering. It arises from accumulated knowledge and experience in rationalizing how enzymes realize their catalytic functions through three-dimensional structural topology (e.g., active-site cavities and tunnels^3–11^), conformational dynamics (e.g., flexibility and domain motion^12–19^), electronic structure (e.g., electrostatics and electric fields^20–25^), and interactions with the surrounding environment (e.g., hydration shell). Such intuition helps protein engineers infer or anticipate the impact of mutations on enzyme activity^26–29^, temperature adaptation^30^, selectivity^31–35^, promiscuity^36–41^, and stability of enzymes^42, 43^. For instance, steric intuition in amine transaminases has led to a design rule for tuning stereoselectivity: by adjusting the volumes of the large and small binding pockets within the active site^3–8^ through site-directed mutagenesis to better match the sizes of the corresponding ketone substituents, one can invert selectivity or expand substrate scope.^33, 44, 45^ Such intuition also strengthens the screening-based, directed evolution approaches for effective function-enhancing mutant discovery. Reetz and coworkers exemplified this through developing the combinatorial active-site saturation test (CASTing) method, which applies targeted saturation mutagenesis to small residue sets and recombines beneficial variants to capture cooperative effects for enhancing activity and selectivity toward non-native substrates.^32, 34, 35, 37, 46–48^ In essence, physical intuition guides the construction of “smart” mutational libraries and facilitates effective enzyme engineering when high-throughput screening assays are not available.

Despite its importance, translating physical intuition into successful enzyme engineering outcomes remains difficult. In practice, most intuitive catalytic principles in enzymology are qualitative rather than quantitative. Gordon Hammes’ famous notion that “For catalysis (of an efficient enzyme), flexible but not too flexible, as well as rigid but not too rigid, is essential” captures a conceptual truth yet provides no guidance for identifying the optimal degree of flexibility.^49^ Even when an intuition has a quantitative foundation, such as the correlation between electric field strength and catalytic rate enhancement,^20–25^ it remains unknown how specific mutations should be introduced to achieve the desired electrostatic environment. As a result, the unique intuition or a stroke-of-genius inspiration from protein engineers always fails to get translated into a “smart” mutational library or a concrete protein engineering strategy. This makes it inevitable to adopt a hypothesis-agonistic approach by screening against a larger library for mutant discovery, leading to greater labor/resource cost and longer enzyme development cycle in most enzyme engineering scenarios.

The pressing challenge facing the entire field of enzyme engineering is how to establish an enzyme engineering paradigm that translates physical intuitions into quantitative, actionable design principles. One promising approach to address this challenge requires high-throughput molecular simulations (htMS), which quantify how mutations alter enzyme structure, dynamics, and energetics to predict their effects on catalytic activity and selectivity. For example, SubTuner leveraged htMS (specifically quantum mechanics (QM) and molecular dynamics (MD) simulations) to translate physical hypotheses (such as optimal transition state binding and electric field stabilization) into quantitative metrics in the *in silico* screening workflow and eventually yield smart libraries (~10 mutants for each task) that constantly achieved ~30% hit rate across three transferase engineering tasks. Similarly, CASCO^50^ leveraged high-throughput MD simulations to quantify substrate positioning dynamics as near-attach-conformation frequencies in the engineering of the enantioselectivity of a limonene epoxide hydrolase; QzymeDesigner^44^ used semi-empirical QM to quantify the substrate binding as free energies in the engineering of the substrate scope of the ω-transaminase from *Chromobacterium violaceum*; and AI.zymes^51^ integrated MD screening into a multi-objective design workflow, using metrics such as electric field strength, ligand flexibility, etc. to prioritize variants for improving the promiscuous Kemp eliminase activity in a ketosteroid isomerase. Yet, despite its demonstrated success, htMS by itself cannot fully bridge the gap between intuition and enzyme engineering practice; its complexity and technical demands often limit accessibility beyond specialized computational experts. As a result, numerous potentially transformative approaches for intuition-guided, molecular modeling-based enzyme engineering have stalled as one-off proof-of-concepts rather than scalable solutions.

**Figure 1.**
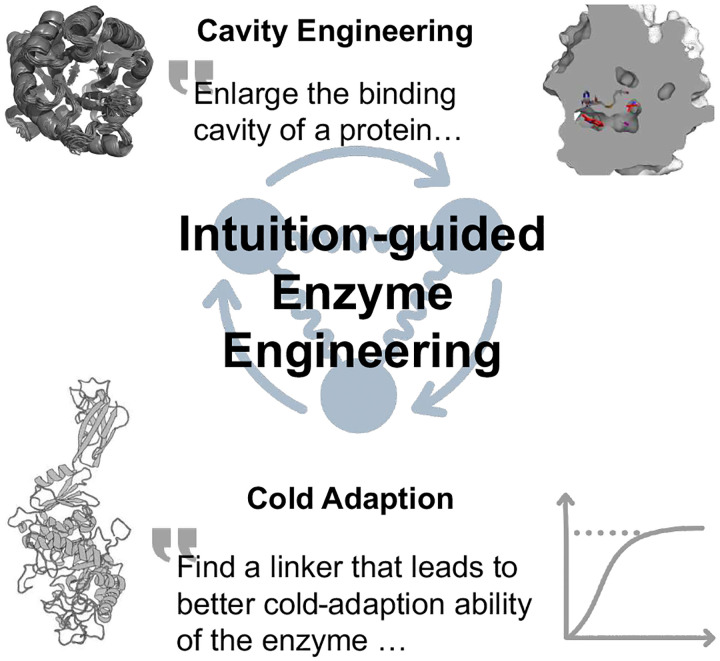
Physical Intuition-guided Enzyme Engineering.

To fully unlock the potential of intuition-guided enzyme engineering, we propose a computational platform that unites large language models (LLMs) with high-throughput molecular simulations. Our solution satisfies two critical needs simultaneously: first, translating qualitative and descriptive intuition into explicit, executable instructions; and second, converting those instructions into automated computational workflows that deliver quantitative, physics-based outputs. To this end, we developed MutexaGPT, an intuition-to-design translator of molecular simulation for enzyme engineering. MutexaGPT is built based on large language model (LLM) agents and works as an open-access web application (https://enzyhtp.app.vanderbilt.edu) that interacts with users in plain English, converts intuition-based requests from users into computational modeling plans, performs high-throughput MD simulations, analyzes the results, and summarizes them into actionable insights for enzyme engineering (such as a “smart” library pinpointing a few mutants). Note that although MutexaGPT ranks and filters enzyme variants based on computed metrics, it is not a predictive AI tool, but instead an integrated system for automation and intuition-translation. Designed for protein engineers and designers with little or no molecular modeling expertise, MutexaGPT is distinct from existing computational chemistsoriented LLM-agentic molecular simulation tools (e.g., MDCrow^52^, NAMD-agents^53^, DynaMate^54^, and ProtAgents^55^) that require users’ domain knowledge to choose theoretical models, design simulation protocols, and interpret results. We achieve so by equipping MutexaGPT’s LLM agents with necessary domain knowledge to guide users through the entire life cycle of high-throughput physics-guided enzyme engineering,^56, 57^ from creating the scientific question to interpreting the results. We demonstrated the effectiveness of MutexaGPT through two cases that showcase how empirical intuition of cavity engineering and statistics-based intuition of cold-adaptation engineering can be converted to physics-based models and guide function-gaining mutant discovery (Figure 1B). Complementary to screening-dominated approaches, MutexaGPT provides a scalable route to operationalize intuition-driven, physics-guided enzyme engineering within the scope of currently implemented metrics and simulations.

## 2. Implementation

### 2.1 The workflow of the multi-agent system in MutexaGPT

**Figure 2.**
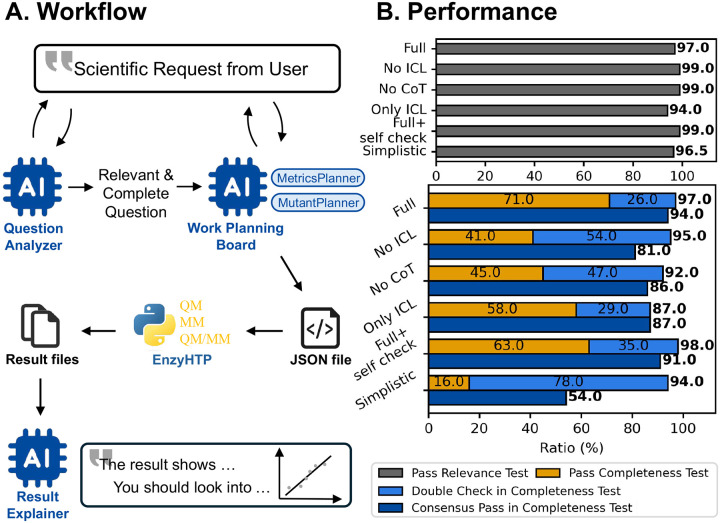
The Design of MutexaGPT. (A) The architecture and workflow of the multi-agent system in MutexaGPT. With a user posting their scientific request, QuestionAnalyzer checks the request for the missing details, provides suggestions to better define the scientific question, helps the user articulate their assumptions, and eventually co-creates a scientifically sound and concrete request. The request is then sent to WorkPlanningBoard (consisting of MetricsPlanner and MutantPlanner) to come up with a molecular modeling plan and seek approval from the user. The user-confirmed plan is then saved as a JSON file, which is sent to the EnzyHTP in the backend for performing the high-throughput simulations. ResultExplainer dissects the result files from the simulation and summarizes them into a concise report for the user. (B) Benchmark of system prompt engineering strategies in the QuestionAnalyzer for relevance (up) and completeness (bottom). (Full = full prompt; No ICL = full prompt with in context learning part removed; No CoT = full prompt with the chain of thought part removed; Only ICL = a prompt containing only in context learning examples; Full + self-check = augmenting the Full prompt with a one-line self-reflection instruction; Simplistic = only contains a sentence of role description and the output format in the system prompt.) The full evaluation results for all agents are summarized in SI.zip/agent_evaluation.

At the heart of MutexaGPT is a multi-LLM-agent system that consists of QuestionAnalyzer, WorkPlanningBoard, and ResultExplainer (Figure 2A). They run, in the web-application backend, to co-create intuition-driven requests, supply necessary domain knowledge, convert the request into physical models, configure computational protocols, and execute the simulations. The users may begin by providing an intuition-driven request (e.g., “help me enlarge the active site”) into the system and expect MutexaGPT to execute relevant computational modeling to guide their enzyme engineering tasks. In its well-versed version, these initial, intuition-driven requests should convey three elements: 1) enzymes of interest, 2) an optional, initial mutation library of interest, and 3) engineering objectives such as enzyme efficiency, stability, etc. Real requests from users, however, are often vague (e.g., the user may just say “mutations” instead of having a specific mutation library in mind), physically inconsistent (e.g., “improve the temperature of the backbone” make no sense), or completely off-topic (e.g., user may misunderstand the specialization of the webapp and ask it to recommend a protein shake brand).

The QuestionAnalyzer agent steps in and starts a conversation with the user to address these problems: for those irrelevant inquiries, it explains the specialization of the webapp and asks the user to resubmit a new inquiry. For the relevant ones, it actively checks for the missing details, helps the user to articulate implicit assumptions, provides suggestions to reframe scientific terms for clarity, and eventually co-creates a scientifically sound and concrete request (see Text S1 for an example dialog). The revised question that satisfies QuestionAnalyzer will then be passed to the WorkPlanningBoard, which is composed of two agents, MetricsPlanner and MutantPlanner. The MetricsPlanner maps the engineering objectives of users to a list of physics-based, EnzyHTP^56, 57^-computable metrics. For example, a complex target such as “drug resistance of mutants” will be decomposed into a combination of enzyme activity, binding affinity, and thermostability, which are then mapped to internal electric field strength, binding free energy, and folding energy. MetricsPlanner functions through a conversation with the user, where it will first suggest combinations of metrics, as well as educate the user with necessary domain knowledge explaining the combination. If the user accepts a suggestion, the MetricsPlanner checks whether all mandatory arguments are present, including: selection masks, restraint definitions, protonation states, and so on. Any omissions are surfaced as explicit queries, each accompanied by a plausible default. This dialogue repeats until every parameter is well defined, at which point the agent compiles a JSON file as the final output in the back end (see Text S1 for an example dialog). In parallel to MetricsPlanner, MutantPlanner executes silently in the background and maps the user’s mutation plan into the EnzyHTP mutation pattern, a powerful syntax defined in EnzyHTP that describes a list of mutants each composed of a list of mutations. Combining the output of MetricsPlanner and MutantPlanner gives the final payload JSON object that is ready to be executed by EnzyHTP autonomously. Notably, MutexaGPT allows users to leverage resources from Advanced Computing Center for Research and Education (ACCRE) at Vanderbilt University for the requested computations. The platform also provides a choice for users to download a folder of prepared input files of all needed simulations and a simple script for configuring the environment, executing them using the users’ local computing resources, and uploading them back to MutexaGPT if needed. Note that to further support reproducibility if users want to publish any data generated from MutexaGPT, in the workflow review page, the website allows users to export the exact EnzyHTP Python script that is executed. This script contains full simulation setup and parameters and can be directly rerun or cited in Methods/Supplementary sections to ensure reproducibility, especially for users without extensive computational background.

Once the simulations are complete, their outputs are collected into a single JSON object that contains (i) the user’s original scientific question, (ii) the mean ± standard-deviation values for every requested metric, (iii) URLs for any downloadable files (trajectory pickles, CSV tables for full results, etc.), and (iv) metadata of the simulation (force field, length of the MD, equilibration assessment, etc.). This JSON becomes the sole input to the terminal agent, ResultExplainer. Acting under the prompt shown in Supplementary Table S2, ResultExplainer handles five top-level goals: (1) translate raw modeling results into concise, domain-relevant takeaways; (2) anchor every explanation to the user’s scientific question rather than to the data in isolation; (3) explain, for each reported metric, what it may indicate biologically or structurally and, equally importantly, what it does not directly measure or prove, thereby reducing the risk of over-interpretation by non-computational users; (4) communicate the confidence and limitations of the analysis in light of the available simulation context, such as simulation length and equilibration information; and (5) prioritize the most relevant variants and recommend follow-up experiments, additional simulations, or data checks (e.g., ResultExplainer can rank/filter enzyme variants based on computed metrics; see Text S10 for examples of ResultExplainer output). By this final message, MutexaGPT closes the loop from a vague, qualitative intuition to experiment-ready design recommendations with molecular level insights (see Text S7 for the design of each individual LLM-agent involved in this workflow).

### 2.2 The performance of agents in MutexaGPT

To assess the performance of the three core agents in MutexaGPT (i.e., **QuestionAnalyzer**, **WorkPlanningBoard**, and **ResultExplainer**), we designed an automated evaluation framework for MutexaGPT (Text S8 and Figure S2). This framework leverages large language models for both generating diverse natural-language test cases (i.e., test-set curator agents) and evaluating agent outputs (i.e., answer classifier agents). Specifically, a group of test-set curator agents was deployed to create test cases tailored to each core (“testee”) agent in MutexaGPT, and all generated cases were manually reviewed for accuracy. For evaluation, a set of classifier agents was used to categorize each testee agent’s natural-language output into predefined labels, which were then compared with manually verified ground-truth labels to determine pass/fail outcomes (see Text S8 for details).

Using this evaluation framework, we benchmarked the performance of the core agents in MutexaGPT. We focus on **QuestionAnalyzer** here as a representative example (Figure 2B) because it is the most sophisticated agent and most directly tied to MutexaGPT’s mission of converting intuition into design. Note that, an LLM agent in MutexaGPT is a large language model (e.g.: GPT-4o^58^, etc.) configured with a system prompt that instruct and regulate its behavior. In this section, “prompt design” refers specifically to the design of the system prompt that governs the agent’s behavior, rather than the natural-language inquiry/input provided by the user. We therefore benchmark how different prompt-engineering choices affect agent performance under the same task definition and curated test set. The performance was evaluated against six different prompt designs (Figure 2B) on the same curated test set of 200 inquiries (100 irrelevant and 100 relevant, with the latter split evenly between fully specified and underspecified cases). The inquiries were generated by the test-set curator and manually reviewed for correctness. Each prompt design was tested across three independent runs, and a design was marked as failing a test if any of the three runs failed.

Evaluation is based on two criteria: relevance (Figure 2B, top) and completeness (Figure 2B, bottom). The relevance metric tests whether **QuestionAnalyzer** correctly classifies user inquiries as relevant or irrelevant. A design is counted as passing only if all three independent runs correctly reject every irrelevant question and accept every relevant one; the reported performance reflects the proportion of designs meeting this standard. The completeness metric assesses whether the agent correctly identifies missing information in under-specified inquiries. We report this metric in three forms: (1) the straight pass ratio, representing designs for which all three runs behave correctly (i.e., the agent asks for exactly the information categories that are actually missing in the inquiry: protein, mutation, and/or property; orange bars); (2) the double-checking pass ratio, a more permissive measure that also counts cases where the agent correctly identifies missing information but unnecessarily re-asks for existing information—behavior that is overcautious yet harmless to downstream automation (light-blue bars, counted together with orange to form the effective pass rate); and (3) the consensus pass ratio, a relaxed criterion that deems a completeness test successful if at least two of the three runs pass, reflecting tolerance toward stochastic errors (dark-blue bars).

The benchmark results show that the *Full* prompt (see Table S2), which contains chain-of-thought^59–61^ (CoT; i.e., the system prompt contains a list of instructions directing the agent to follow a logic chain) scaffold and seven carefully selected in-context learning^62^ examples (ICL; i.e., examples, as input and output pairs, are provided in the system prompt, also known as few-shot learning), emerges as the best performing design. It accepts or rejects queries correctly in 97.0% of cases, achieves an 71.0% strict completeness pass rate, and climbs to 97.0% once double-checks are counted; its consensus robustness stands at 94.0%. Removing the ICL snippets (*No ICL*) leaves the relevance pass rate almost unchanged (99.0%) but cuts the strict completeness pass rate to 41.0%, because the model becomes uncertain and compensates with many redundant queries (54.0% in double-check rate). Suppressing the explicit reasoning scaffold instead (*No CoT*) has a similar although slightly less severe effect: strict completeness pass falls to 45.0%. The *Only ICL* prompt, which only contains the seven examples and a single-sentence role description, recovers some ground (58.0% strict completeness pass rate) yet remains less stable. Augmenting the *Full* prompt with a one-line self-reflection instruction (*Full + self-check*) did not translate into a net win. Although the effective pass rate remains high (98.0%), the strict completeness pass rate falls by roughly eight percentage points and the proportion of redundant double-checks grows, as well as pulling the consensus robustness down from 94.0% to 91.0%. In other words, the extra self-check mostly prompts the agent to question information that is already complete, offering little practical benefit while adding conversational noise. At the opposite extreme, a minimalist specification (*Simplistic*), which only contains a sentence of role description and the output format in the system prompt, still rejects irrelevant questions (96.5%) but collapses on strict completeness (16.0%), succeeding in barely half the cases even though the looser consensus pass is measured (54.0%). Error analysis of the result of the Full QuestionAnalyzer shows that relevance failures are exclusively false negatives, primarily arising from overly vague inquiry (e.g., “What effect do ligands have on proteins?”), while effective-pass completeness failures are rare (3/100) and mostly associated with marginally ambiguous property definitions (e.g.: “flexibility of …”) rather than systematic inability to identify incomplete inquires.

Three design lessons follow. First, CoT and ICL contribute independently and additively to the agent’s ability to solicit precisely the missing information, removing either one reduces the performance by 25–30 percentage points. Second, a cheap self-check clause provides an easy safety net against occasional lapses. Third, a blanket “self-check before replying” clause is not an automatic upgrade. In our hands, it lowered strict completeness and inflated redundant follow-ups. Third, minimal prompts are insufficient for complex reasoning. The Simplistic design, removing CoT and examples, collapsed to one-third completeness pass rate and showed the highest rate of redundant clarifications, underscoring the necessity of structured guidance for complex tasks (e.g., CoT, ICL, etc.). Without them, the model defaults to hesitation and repetitive questioning instead of reliable information gathering. Note that, while the Full prompt design performs better in our context, a richer system prompt carries a potential downside: it improves in-domain precision by constraining the agent, but also reduces flexibility on atypical or out-of-distribution requests by anchoring behavior to the included examples and interaction schema.^63, 64^

Besides **QuestionAnalyzer**, every agent in the MutexaGPT workflow is subjected to its own benchmark similar to the one described above, albeit with a single, production-ready prompt rather than the full suite of design variants explored above (Text S8). For MetricsPlanner, the test-set curator generates inputs (that match the format of the QuestionAnalyzer’s final output, with 100 cases asking about properties that can map to one of our supported metrics, and 100 that are not) from ground-truth metric/MD-model labels, and a case passes only when the agent’s recommended candidates contain the labeled metric-model pair or inform user when none of the supported metrics can correspond to the inquiry. For MutantPlanner, curator-agent generated inputs contain a balanced set of unrestricted, pocket, protein-protein interface, distal (relative to the pocket) mutational libraries, and designated site mutations (10 for each, 50 in total). The correctness is judged by whether the generated EnzyHTP mutation pattern is semantically equivalent to the labeled library (i.e., they are parsed by EnzyHTP and directly compared for equivalence). For ResultExplainer, a set of JSON payloads are curated by a curator agent (containing “scientific question”, “metrics”, “results”, “downloadables”, and “metadata”; 100 in total). The outputs of ResultExplainer are evaluated by two judge agents, one assessing how well the output aligns to the scientific question from the input and the other assessing interpretationboundary discipline (i.e., avoidance of overclaiming beyond the provided data), and overall pass requires satisfying both criteria. Full benchmark inputs, pass definitions, and per-run outputs are provided in Text S8 and SI.zip/agent_evaluation.

The results show that the prompt design for each agent is highly effective in their respective roles: for **WorkPlanningBoard**, MetricsPlanner on average passes 91% of its test cases, MutantPlanner achieves a 95% success rate; **ResultExplainer** met all evaluation criteria in 99% of the test cases. While not directly comparable, these performance levels are strong in the broader context of LLM benchmarks for scientific tasks (for reference, recent scientific-discovery benchmarks^65^ typically report only 0.60–0.75 question-level accuracy across domains, while the best model in a question-clarification benchmark^66^ reaches a maximum success rate of 60.1%). One can find details in Text S9 for error analysis of each agent, and SI.zip for spreadsheets that contain the test input, ground truth label, agent’s output, the judge agent’s output, and the pass/failure of every test case of each agent in MutexaGPT.

## 3. Results

This section presents two MutexaGPT-enabled enzyme-engineering campaigns that translate intuition (written in plain-English) into physics-based models, molecular simulation workflows, and eventually actionable design recommendations. First, a cavity-engineering task uses high-throughput MD ensemble analysis to pinpoint mutations that enlarge an active site, improving accommodation of bulkier, non-native substrates. Second, a cold-adaptation engineering task employs high-throughput MD to design linker variants that enhance enzyme activity at low temperatures; the resulting design was also experimentally validated. In both cases, a shared obstacle that often deters enzyme engineers from translating their intuition into design is the absence of quantitative and actionable guidance and the steep expertise required for high-throughput physics-based modeling. Below, we demonstrate how MutexaGPT addresses these challenges and leads to new scientific discoveries and understandings.

### 3.1 MutexaGPT for enzyme cavity engineering to modify substrate specificity: translation of empirical intuition into mutation design.

In the first case, we asked MutexaGPT to engineer the cavity of a halide methyltransferase toward catalyzing bulkier substrates for group transfer. Cavity engineering targets the shape and volume of the interior voids of an enzyme, and designs or screens mutations accordingly to modulate the enzyme’s functions and properties. An empirical intuition in cavity engineering is “larger cavity allows the enzyme to accommodate larger substrates with enhanced promiscuity”. Although static active-site structures have enabled successful cavity-guided engineering in several systems,^32, 33, 44, 67, 68^ such intuition is difficult to apply broadly. Simple mutations that appear to enlarge a cavity often fail because conformational dynamics can drastically reshape the pocket. As a result, most cavity engineering efforts still rely on screening of a large mutational library, consuming substantial experimental resources.

**Figure 3.**
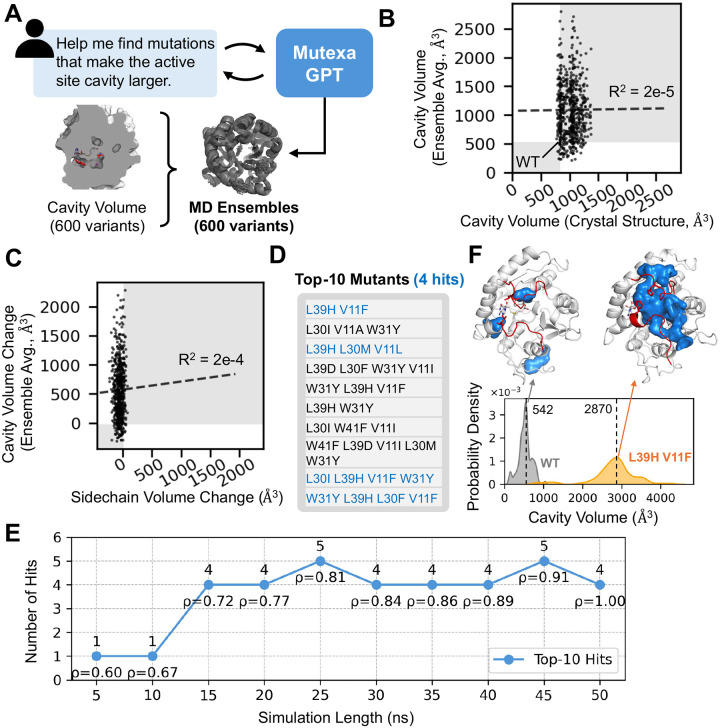
An overview of MutexaGPT-guided cavity engineering. (A) Workflow overview: the user sent a plain-English request; MutexaGPT asked for more information and discussed with the user about the modeling plan; MutexaGPT performed high-throughput MD simulations and calculated cavity volumes for the library of 600 mutants. (B) Correlation between cavity volumes calculated from direct geometric perturbation of static crystal structures (see Method for details) and those calculated from MD ensemble averages; both for the library of 600 mutants. (C) Correlation between the change in side-chain volume versus the change in ensemble average cavity volume for the library of 600 mutants. (D) MutexaGPT’s final list of 10 mutants with the largest predicted cavity volumes. Experimentally confirmed hits are highlighted in blue; these mutants show activity with bulkier substrates^69^. (E) The top-10 hit-rate plot against the MD simulation length of each mutant. The spearman correlation coefficient (ρ) is noted under each data point. (F) Cavity volume distribution of a top-ranked mutant, L39H/V11F against that of the WT. The probability distribution of the mutant’s cavity volume (L39H/V11F, shown as orange) is shifted to a much larger value (~2870 Å^3^) compared to the wild-type (WT, shown as grey) enzyme (~542 Å^3^). Representative structures from the distribution center (dotted line, labeled) show how mutations (stick, red) induce a conformational change in an active site loop (cartoon, red), leading to a significantly larger and more open active site (surface, blue). The kernel density distribution is fitted using the *gaussian_kde* function from the *scipy* package. The fitting data are obtained from the cavity volume calculations of 100 snapshots sampled from the MD trajectory.

MutexaGPT allows the user to translate such intuition into concrete physics models and testable designs through high-throughput MD simulations. The user can start a cavity engineering task by sending a plain-English request in the chat. In our demo, the user sent “*Help me find mutations that make the active site larger*” (Figure 3A). MutexaGPT evaluated the request and found missing scientific details that were essential to the subsequent modeling (i.e., a target enzyme structure and a list of desired mutations). As such, it asked the user to provide a PDB file of the target enzyme and a description of mutations that the user wants to include (see full conversation log in Text S1). To address this request, the user uploaded the PDB structure of the wild-type halide methyltransferase and supplied details of a mutation library (i.e., 600 combinatorial mutations from five sites, see Text S1) previously constructed for this enzyme by Schülke et al.^69^ The experimental sequence–activity data derived from the library screening against five substrates of diverse sizes allow us to investigate how changes in cavity volume influence enzyme activity later on. In real-world applications, users can initiate their protein engineering campaigns with MutexaGPT by simply describing their desired mutation library, such as “1000 random mutations across the enzyme”, “single-point saturation mutations within 5 Å of the heme cofactor”, and so on.

After verifying that the user’s input contained all necessary information to proceed with the modeling, MutexaGPT planned a relevant molecular simulation task: calculating the cavity volume of the active site of each mutant from MD-sampled conformational ensemble of the holo-enzyme (see Method section for full computational details). With the user’s approval, the backend executed the corresponding computational workflow. For each of the 600 multi-point mutants in the library, MutexaGPT generated a 50 ns MD trajectory and analyzed the distribution of active site volumes across the trajectory. The length of each MD simulation is an option that the user can specify in the frontend before submitting the job. We used 50 ns here to mimic a realistic scenario where most enzyme engineers do not have access to extensive computational resources and need to balance computational cost and sampling adequacy. Even with this moderate MD simulation length, it would still demand 30 μs sampling (i.e., 600 mutants × 50 ns) to cover the entire mutation library.

Eventually, MutexaGPT addressed the user’s initial request, “*Help me find mutations that make the active site larger*”, by ranking all 600 variants according to their ensemble-averaged cavity volumes and identifying the 10 candidates showing the greatest increases in volume (Figure 3D, see screenshot of the Summary page in Figure S3), thereby turning the original empirical intuition into testable mutation designs. To validate these designs, we compared MutexaGPT’s top-ranked mutations with experimentally characterized variants reported by Schülke et al.^69^ Among the ten top-ranked variants, four were confirmed experimental hits based on Schülke et al.’s prior study, yielding a 40% hit rate and around 4-fold improvement over the baseline (see Text S12 for details about the baseline). To investigate whether 50 ns MD simulations are sufficient for cavity-based smart library construction, we systematically examined how simulation length influences hit enrichment and ranking fidelity (Figure 3E). Very short simulations (5–10 ns) fail to enrich hits beyond the baseline expectation (i.e., ~1 hit), whereas simulations of 15 ns or longer consistently identify at least 4 hits, corresponding to about a 4-fold enrichment. Meanwhile, the Spearman rank correlation with the 50 ns reference increased monotonically with simulation length, indicating progressively more stable cavity-volume estimates. Together, these results show that moderate-length (i.e., 15 ns or longer) MD simulations provide reliable quantitative guidance for mutation prioritization in cavity-engineering campaigns.

A case study of the top mutant, L39H/V11F, illustrates how mutations induce a drastic upshift in the active site’s cavity volume distribution compared to the wild-type (WT) enzyme (Figure 3F), thereby reshaping its substrate preference. The cavity volume for the most probable WT conformation is approximately 542 Å^3^, corresponding to a closed and compact active site. In contrast, the L39H/V11F mutant’s cavity volume distribution is centered at a much larger value of ~2870 Å^3^, reflecting a significantly larger and more accessible cavity. This transition from a closed to an open cavity state is primarily driven by the altered conformational dynamics of two key active site loops (residue 1–11 and residue 36–46; highlighted in red, Figure 3F). This cavity expansion in the active site directly translates to improved substrate promiscuity toward a variety of substrates, especially the sterically bulkier ones. While the WT is evolved by the nature to catalyze the methyl transfer from iodomethane to S-Adenosyl-L-homocysteine (SAH), L39H/V11F showed an substantially increased specific activity (relative to the WT) for bulkier substrates^69^ such as iodoethane (10-fold activity improvement), 1-iodopropane (49-fold), 1-iodobutane (50-fold), (iodomethyl)cyclopropane (50-fold), and (2-iodoethyl)benzene (29-fold). These results demonstrate that MutexaGPT can effectively convert intuition into physics models and design through high-throughput molecular modeling, identifying function-enhancing mutations that alter substrate preference.

Given the computational expense of MD simulations, an important question arises: is MD truly necessary for quantitative cavity engineering, or can the ensemble-averaged cavity be reliably inferred from a single static structure as a shortcut? Using the MD trajectories generated by MutexaGPT for 600 variants, we first examined the correlation between the ensemble-averaged cavity volume from MD and that calculated from a single static PDB structure (with optimized side-chain rotamers), assessing whether static structures can adequately serve as surrogates for dynamic ensembles in computational enzyme engineering. The results show no correlation (R^2^ ≈ 2×10^−5^) between the static and MD-averaged volumes, where the cavity volumes derived from MD trajectories distribute significantly broader than from static PDB structures (standard deviation: 488 Å^3^ vs. 145 Å^3^, Figure 3B). This lack of correlation originates from the presence of cryptic cavities that emerge only in the MD ensemble through conformational fluctuations. These transient pockets remain closed or buried in the crystalline state represented by a single static PDB structure. As a second test, we evaluated another common cavity engineering shortcut that estimates the magnitude of cavity expansion in the mutant from the net decrease in side-chain volume due to point mutations. Specifically, we tested the correlation between the change in side-chain volume and the change in ensemble-averaged cavity volume derived from MD trajectories. This approximation also failed to show any meaningful correlation (R^2^ ≈ 2×10^−4^; Figure 3C), indicating that cavity change originates from collective and dynamic rearrangements of the protein active-site structure rather than from simple steric subtraction, which explained why the traditional qualitative intuition-based design often fails. Together, these two tests demonstrate that simple approximations cannot capture the intrinsic conformational plasticity of enzyme cavities, underscoring the necessity of physics-based modeling, specifically, MD simulations for predictive cavity engineering.

To further test whether MutexaGPT remains useful when prior information about mutational hotspot is minimal, we designed a test where the mutation library is selected solely based on a distance cutoff from the substrate. In this new case, the user starts by asking “help me find mutations that make my enzyme faster for an non-native substrate ethyl iodide.” and during the user’s conversation with the QuestionAnalyzer, the mutation library was defined by user as “I want to explore all single point mutations with in 4A of ETI.”, where ETI is the 3-letter name of the target substrate (i.e., ethyl iodide) in user’s PDB files. After the conversation, MutexaGPT plans a workflow that calculates the folding free energy and the electric field strength projected to the breaking C–I bond for each enzyme mutant. With the simulation data, MutexaGPT summarizes the results by filtering candidates with folding free energy and ranking by electric field, yielding a top-10 mutant list, of which 3 mutants are experimentally active in a previous screening reported by Tang *et al*.^70^, corresponding to a hit rate of 30%. Notably, this 30% value serves a lower bound because 4 mutants on this list were not experimentally characterized (not labeled in our test) and could be a hit. Overall, this low-prior-knowledge setup results in a similar prediction performance to the cavity engineering example, supporting MutexaGPT’s ability to convert broadly-defined, less-informed design intent into actionable screening priorities.

### 3.2 MutexaGPT for amino acid linker engineering to enhance cold adaptation of bidomain enzymes: translation of statistical intuition into mutation design.

**Figure 4.**
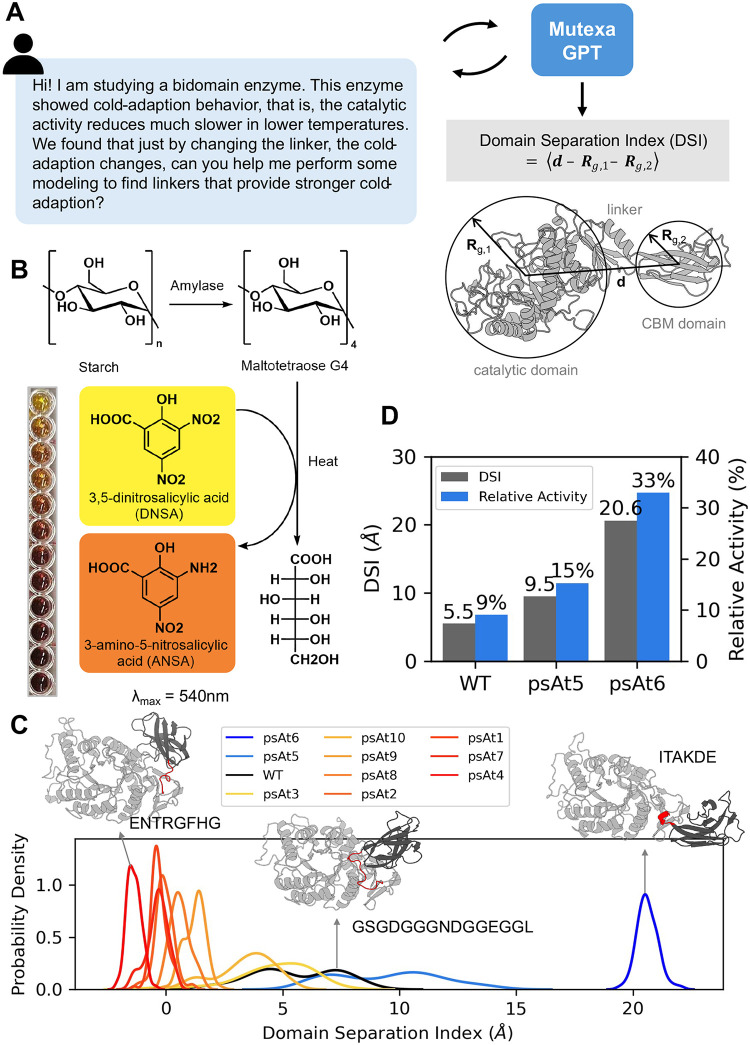
MutexaGPT-guided linker design for cold-adapted bidomain amylases. (A) Workflow overview: the user sent a request in plain text; MutexaGPT collected missing information from user, discussed and planned the modeling, performed MD for WT and 10 linkerreplaced variants, and calculated the domain separation index (DSI). DSI is defined as ⟨d − R_g,1_ − R_g,2_⟩ over the MD ensemble, where d is the distance between the two domain centroids and R_g,1_/R_g,2_ are the radii of gyration of the two domains, respectively. Unit: Å. (B) The experimental assay for the amylase activity: specific activity is measured via the 3,5-dinitrosalicylic acid (DNSA) reducing-sugar assay; cold-adaptation is evaluated as the “relative activity” = activity at 0 °C divided by activity at 45 °C. (C) The DSI distribution of the *Pseudomonas saccharophila* amylase wild type and its ten linker variants. For variants with the largest (psAt6) and smallest (psAt4) average DSI and the wild type, a representative structure of the distribution peak is attached. (grey: catalytic domain, red: linker, dark grey: CBM domain) The linker sequences are noted next to each structure. Note that the wild type shows two equally distributing peaks, see Figure S10 for representative structures of both peaks. The kernel density distribution is fitted using the *gaussian_kde* function from the *scipy* package. The fitting data are obtained from the DSI calculations of 100 snapshots sampled from the MD trajectory of each variant. (D) The experimental results of MutexaGPT suggested variants: two linkers (psAt5, psAt6) predicted to have higher DSI than WT also showed increased relative activity in experiments. (See full experimental and computational detail in Method Section)

In the second case, we integrated MutexaGPT with experimental assays to engineer cold-adapted variants of a bidomain amylase by redesigning its interdomain linker, aiming to enhance enzymatic activity at low temperatures. Cold-adapted enzyme engineering, the process of identifying enzyme variants that maintain high activity at colder conditions (e.g., 0–15 °C), is crucial for low-temperature laundry^71^, food processing^72, 73^, bioremediation^74, 75^, and single-cell sequencing^76^, serving as a cornerstone for modern biomanufacturing and biotechnology. From MD modeling, we uncovered a “statistical intuition” that suggests the correlation between increased interdomain separation and enhanced cold adaptation in bidomain amylases and cellulases.^30, 77^ This separation is quantified by the spatial proximity between the carbohydrate-binding module and the catalytic domain, defined as the domain separation index (DSI, Figure 4A). Guided by this statistical intuition, MutexaGPT can help user establish a closed-loop computational–experimental workflow. In this workflow, MutexaGPT identified linker variants of a maltotetraose-forming amylase (Figure 4B) predicted to exhibit increased DSI, and these variants were subsequently tested for enhanced cold adaptation through enzyme activity assays.

The user initiated the task by sending the following request to MutexaGPT: “*I am studying a bidomain enzyme. This enzyme showed cold-adaptation behavior, that is, the catalytic activity reduces much slower at lower temperatures. We found that just by changing the linker, the cold-adaption changes, can you help me perform some modeling to find linkers that provide stronger cold-adaption?*”. Similar to the first case, MutexaGPT asked for clarification of the target enzyme by requesting PDB files and the linker sequences of interest. The user provided the AlphaFold2-predicted structure of the mesophilic *Pseudomonas saccharophila* amylase (psA), as well as 10 psA variants with different linker sequences selected from natural peptide linkers in LinkDB^78^ and SynLinker^79^ databases based on the linker topology from our previous study (see Table S3 for linker sequences and Text S13 for the selection workflow). With this information, MutexaGPT devised a plan to identify linker variants predicted to exhibit higher DSI values than the wild type by performing 50 ns MD simulations for each candidate. Upon the user’s confirmation, MutexaGPT executed the simulations, analyzed the trajectories, and summarized the DSI results for all variants (see Text S2 for the full dialogue; Table S3 for the list of DSI values).

The DSI distributions for all ten linker variants (psAt1–psAt10) display a distinct pattern (Figure 4C). We observe that variants with extreme DSI values tend to adopt more stable interdomain conformations. psAt6, for example, shows the highest average DSI (20.6 Å) with a very narrow distribution (variance: 0.2 Å), while psAt4, psAt7, psAt1, psAt2, psAt8, and psAt9 cluster near 0 Å with similarly tight distributions (variance: 0.1–0.2 Å). In contrast, the wild type and variants with moderate DSI values, including psAt3, psAt10, and psAt5, exhibit much broader DSI distributions (variance: 1.4–4.8 Å). These patterns arise from distinct linker scaffolds. In high-DSI variants, the two domains are well separated and connected by a linker that predominantly adopts an α-helical conformation (Figure 4C, right), restricting relative domain motion. In low-DSI variants, the two domains pack closely and form extensive interdomain contacts, likewise limiting flexibility (Figure 4C, left). By contrast, variants with intermediate DSI values, as well as the wild type, feature a more disordered linker that samples diverse orientations between the domains, leading to broader DSI distributions.

Two variants, psAt5 (blue) and psAt6 (deep blue), center around 9.5 Å and 20.6 Å, respectively, significantly exceeding the DSI range of the wild type (around 5.5 Å). These two variants were therefore suggested by MutexaGPT for further experimental characterization (Figure 4B). We then experimentally assessed the specific activity of these two variants at different temperatures by measuring the amount of reducing sugar produced from starch using a 3,5-dinitrosalicylic acid (DNSA) assay^80^ (Figure 4B). The magnitude of cold adaptation was assessed by calculating the relative activity, defined as the ratio of the enzyme’s activity at 0°C to its activity at 45°C. The experimental results (Figure 4C) show that psAt5 and psAt6 exhibit 1.7-fold and 3.7-fold increases in relative activity, respectively, compared to the wild-type enzyme, indicating that both variants involve enhanced cold adaptation (See Table S4 for activities under each temperature). Together, these results validate MutexaGPT’s capability to seamlessly convert statistical-intuition into physics-based models, integrate with experimental workflows, and conduct physics-guided engineering of cold-adapted enzymes.

## 4. Discussion

In this work, we developed MutexaGPT as an intuition-to-design translator for enzyme engineering. The challenge of intuition-to-design conversion was addressed by integrating human intelligence with machine intelligence through a multi-LLM-agent system, where MutexaGPT assists enzyme designers and engineers to reframe their intuition-driven ideas into physics-based structure-function hypotheses via the QuestionAnalyzer agent, converts the hypothesis into a high-throughput molecular simulation task via the MetricsPlanner and MutantPlanner agent from the WorkPlanningBoard, operates on the modeling by calling our high-throughput molecular modeling toolkit EnzyHTP, and eventually analyzes the results using the ResultExplainer agent. As such, the user can convert their plain English requests into a “smart” library of enzyme mutant designs through the MutexaGPT web application. Notably, while MutexaGPT ranks and filters enzyme variants based on computed metrics, it is not a predictive AI tool, but instead an integrated system for automation and intuition-translation.

We evaluated the performance of MutexaGPT’s core agents (i.e., QuestionAnalyzer, WorkPlanningBoard, and ResultExplainer) using an automated, LLM-based benchmarking framework. Curator agents generate diverse, manually verified natural-language test cases, while classifier agents assess outputs against ground truth to quantify relevance and completeness. Using QuestionAnalyzer as a representative and most intuition-critical example, we found that prompt designs combining structured reasoning scaffolds and in-context examples consistently outperform minimal or heuristic prompts, which suffer from incomplete information gathering and redundant clarification. Similar evaluations show that WorkPlanningBoard and ResultExplainer also achieve high reliability with their production-ready prompts. Overall, these assessments demonstrate that MutexaGPT’s agent design and prompt strategies support the translation of user intuition into actionable decisions.

Eventually, we demonstrated MutexaGPT in two enzyme engineering case studies. First, we engineered the cavity of a halide methyltransferase to accommodate bulkier substrates. By converting a “cavity”-related empirical intuition into a physics-guided design, MutexaGPT yielded a smart library that achieves 40% hit rate with around 4-fold improvement over the baseline. Second, we engineered a bidomain amylase to enhance its activity under low temperature conditions. By translating a statistical structure-function intuition into physics-guided design, MutexaGPT assisted the user to identify amylase variants that exhibit up to 3.7-fold increase in cold adaptation at 0 °C in our experimental validation. Complementary to existing screening-heavy approaches, we believe MutexaGPT will enable a new paradigm for enzyme engineering where human creativity is seamlessly integrated with computational simulation to make new discoveries and designs.

How does MutexaGPT work beyond the two case studies? Currently, MutexaGPT supports high-throughput calculations of a list of enzyme function-related metrics: electric field strength, substrate positioning index (SPI), active site RMSD, MM-PB/GBSA ligand binding energy, relative folding free energy, cavity volume, and domain separation index (DSI). Beyond the non-native substrate accommodation (metric: cavity volume) and cold adaptation (metric: DSI) as demonstrated in the current study, this full metrics list can be applied to a large variety of enzyme engineering tasks. For example, the cavity volume metric guides the engineering of enantioselectivity.^33, 44, 45^ Electric field strength can guide the engineering of rate enhancement for enzymes that catalyze reactions involving a substantial change of charge and polarity state from the reactant state to the transition state.^20–25^ MMPB/GBSA ligand binding energy can guide the engineering of enzyme with enhanced binding affinity to the substrate or reduced product inhibition.^81^ SPI can guide the engineering of catalytic efficiency for enzymes where precise substrate positioning is critical for catalysis.^82^ Relative folding free energy can be used to guide the engineering of enzyme stability. Combining these metrics is expected to address more complex challenges, such as studying drug resistance mutations. Note that a practical limitation of MutexaGPT also comes from this list of computable metrics. If a property of interest from the user cannot be mapped to a currently supported EnzyHTP-computable metric, MutexaGPT cannot produce a physically grounded simulation answer and should instead return an explicit unsupported-property notice. Nevertheless, MutexaGPT is designed to be extensible, where new metrics can be easily (by changing just two files if they are supported by EnzyHTP) added in the future to support more enzyme engineering tasks. (One can refer to Table S1 for currently supported metrics; We should note that the underlying EnzyHTP engine is developed with interface modules to common software such as Amber^83^ for trajectory analysis, Gaussian^84^/xTB^85^ for quantum mechanics, Multiwfn^86^ for wavefunction analysis, Rosetta^87^ for biophysics, etc. and thus inherits the metrics-support-boundary of these software) Overall, we envision MutexaGPT can be applied to a wide range of enzyme engineering tasks by leveraging the existing metrics and adding new metrics in the future.

Finally, we would like to discuss MutexaGPT’s level of autonomy as an AI agent. Gao et al.^88^ summarized the three levels of autonomy in AI agents: *assistant level*, which works with restricted range of tools (e.g., web search, docking, etc.) and relies on domain experts to define scientific hypotheses, propose theoretical models, and design specific tasks; *collaborator level*, which collaboratively refine hypotheses with human, design computational tasks, and interpret results; and *scientist level*, which develops and extrapolates scientific hypotheses beyond the scope of prior research, autonomously designs and performs experiments, synthesize concepts beyond summarizing the results, and eventually make new scientific discoveries all by itself. Existing LLM-agentic molecular simulation tools mostly operate at the assistant level, designed to streamline the molecular modeling process for domain experts.^88^ In contrast, MutexaGPT is featured by its functionalities that one would typically expect from a collaborator, such as refining intuition into physics-based hypotheses, executing the htMS, and generating design recommendations. For protein engineers with little-to-no experience in computational modeling, MutexaGPT, while remaining an assistant-level system, takes a step toward becoming a collaborator-level agent for enzyme engineering.

However, the level of hypothesis refinement is still limited to correcting non-scientific statements, retrieving missing information for a vague request, and mapping general properties of interest to specific computable metrics. Presumably, a complete collaborator level agent should also be able to proactively generate alternative mechanistic hypotheses rather than merely refining user-proposed ones, design and adapt multi-step computational and experimental workflows to discriminate among these hypotheses, integrate heterogeneous evidence across modalities to revise or reject initial ideas, and autonomously propose new lines of inquiry when the accumulated data reveal gaps or inconsistencies. Such limitation comes from the limited reliability and robustness of current foundation models for complex, open-ended scientific reasoning, the lack of mature methods for uncertainty quantification and self-evaluation, and the scarcity of standardized, high-quality multimodal datasets and workflows needed to safely couple *in silico* molecular modeling with real-world experimentation. With the unprecedented evolving speed in AI technology and foundation models, we expect these limitations to be progressively alleviated and MutexaGPT will move closer to a genuinely collaborator-level agent.

## 5. Method

### 5a. Computational Method

#### Protein structure preparation.

In the cavity engineering use case of MutexaGPT, the uploaded wild-type structure of *Aspergillus clavatus* methyltransferase (*acl*-MT) is obtained from the AlphaFold Protein Structure Database (UNIPROT ID: A1CIS5). The S-Adenosyl-L-homocysteine (SAH) cofactor is placed in the structure referencing the binding position in the crystal structure of a homologous anion methyltransferase from *Arabidopsis thaliana* (PDB ID: 3LCC). In the cold-adaptation engineering use case of MutexaGPT, the uploaded wild-type structure of *Pseudomonas saccharophila* amylase (psA) is obtained from our previous study^30^, which used AlphaFold2^89^ via the ColabFold^90^ server to predict the protein structure from its sequence. The sequence and parameters used for prediction are listed in Table S5 and Table S6. The rest of the structure preparation procedures, including determining the protonation states of titratable residues, generating structures of enzyme mutants, etc., are planned and performed automatically by MutexaGPT, powered by EnzyHTP^56, 57^ in the backend. (via the *protonate_stru(), mutate_stru(), assign_mutant()*, etc. APIs in EnzyHTP. See full API parameters and underlying algorithms in https://github.com/ChemBioHTP/EnzyHTP-GPT/blob/main/flask-server/templates/slurm_run/md_main_script.py and https://github.com/ChemBioHTP/EnzyHTP)

#### Molecular dynamics.

All MD simulations in this study are performed autonomously on the MutexaGPT server. In the server backend, EnzyHTP is used to perform the MD simulations via the *equi_md_sampling()* API. This API uses a default force field combination of AMBER ff14SB^91^ for the protein, TIP3P^92^ for water, and the generalized AMBER force field^93, 94^ for ligands with the AM1-BCC^95^ charge model. This API, by default, solvated the enzyme in a periodic truncated octahedron box with a 10 Å buffer of water and were neutralized with Na^+^ or Cl^−^ counterions. The production time was set to 50ns via the web GUI in the frontend of MutexaGPT. In the *equi_md_sampling()* API, via the underlying interface with AmberMD^96^, each 50ns MD production run is preceded by 1) 20000 steps energy minimization of the entire simulation system using the steepest descent method for the first half and the conjugate gradient method for the other half. 2) 50 ps heating from 0 to 300 K with constant volume and the backbone C_α_, C, and N of the amide group restrained with a weight of 2 kcal·mol^−1^·Å^−2^. 3) 500 ps equilibration at 300K and 1 atm with the same backbone restraint. 4) 500 ps equilibration at 300K and 1 atm without the backbone restraint. Chodera automated equilibration detection scheme^97^ is used to analyze the equilibration statistics in the backend and the results are reported by ResultExplainer in the final Summary page. All MD simulations were performed with a time step of 2 fs with the SHAKE^98^ algorithm applied. The API by default uses the Langevin thermostat^99^ and Berendsen barostat^100^ to control the temperature and pressure, respectively. (Source code: https://github.com/ChemBioHTP/EnzyHTP-GPT/blob/main/flask-server/templates/slurm_run/md_main_script.py)

#### Cavity volume calculation.

All ensemble cavity volume calculations in this study are performed autonomously on the MutexaGPT server. In the server backend, MutexaGPT calls the *ensemble_cavity_volumes()* API from EnzyHTP when the cavity volume calculation is needed. The *ensemble_cavity_volumes()* API allows the upstream calls to specify the target cavity using a list of composing residues, the containing ligand, or an existing Cavity() object. For *acl*-MT, the active site cavity is defined as composed of residues 7, 11, 27, 40, 41, 43, 165, 168, 169, 199, 210, 211, and 289 (SAH). The API then tracks this cavity along the MD trajectory and obtain the cavity mesh as a *pyvista.PolyData* object^101^, for each frame, using the default, Voronoi-diagram-based, MOLE2 algorithm via the corresponding MOLE2^102^ interface in EnzyHTP. The final cavity volume is obtained through the *.volume* attribute of the mesh object. For the static structures cavity volumes, we used the same protocol in EnzyHTP but with the static mutant structure as input. These structures are generated by replacing the mutating side chains in the wild-type structure and search through a rotamer library to find the optimal side-chain position. (using the *mutate_stru()* function in EnzyHTP)

#### Domain separation index (DSI) calculation.

All DSI calculations in this study is performed autonomously on the MutexaGPT server. In the server backend, MutexaGPT calls the *dsi()* API from EnzyHTP when needed. For each MD trajectory, the *dsi()* API calls cpptraj to calculate, for each frame, the distance between the center of mass of the designated domains and the radius of gyration of each domain (*R*_*g*_). Then DSI = domain distance – (R_g,1_ + R_g,2_).

#### Configurations of the large language model (LLM).

All LLM agents used in this study is powered by the OpenAI Python API, specifically, the *Responses* API for the native agentic tool call support from OpenAI. The LLM version is set to *gpt-4o-2024-11-20* for the consistent performance besides for ResultExplainer which is set to *gpt-5.4* for large context window. (temperature: 1.00; Top P: 1.00) Note that users can also choose the LLM model they want to use on the MutexaGPT website. (current options include: GPT-4o, GPT-5.4, Qwen3.6 Plus, MiniMax-M2.5, GLM-5, Kimi K2.5, GLM-4.7, Deepseek v3.2, Gemma 4 26B A4B IT, Gemma 4 31B IT) When using the OpenAI models, users need to provide their own OpenAI API keys. Each LLM agent is equipped with tools that allows them to interact with system in the backend and the user experience in the frontend. (see design details in Text S7 and system prompts in Table S2)

### 5b. Experimental Method

#### Bacterial Strains, Plasmids and Cloning.

Genes encoding psA and psA variants (psAt5 and psAt6) were synthesized by Integrated DNA Technologies (Morrisville, NC). We amplified the synthesized genes through polymerase chain reaction using Q5 High-Fidelity Master Mix (New England Biolabs). We assembled the amplified genes and pET-22b vector using Gibson Assembly Master Mix (New England Biolabs). We transformed the recombinant plasmids into Escherichia coli JM109 and E. coli BL21 for DNA manipulations and protein expression, respectively.

#### Protein Expression.

A single colony from the transformed cells was inoculated into Luria-Bertani medium (5 mL) supplemented with ampicillin (50 μg mL−1) and grown at 37 °C overnight with agitation at 220 rpm. The overnight culture (2 mL) was then inoculated into Terrific-Broth medium (200 mL) supplemented with ampicillin (50 μg mL−1). The culture was incubated at 30 °C with agitation at 160 rpm for 72 hours. After incubation, the cells were removed by centrifugation at 10,000 × g for 30 minutes at 4 °C, and the supernatant, which contained crude enzymes, was collected for the thermal adaptation characterization.

#### Thermal Adaptation Characterization.

The hydrolytic activities of amylases were measured by determining the reducing sugars released from the hydrolysis of starch using the 3,5-dinitrosalicylic acid (DNS) method^80^. The reaction mixture containing 0.2 mL of the enzyme and 1.8 mL of 1% (w/v) potato starch (Fisher Scientific) in 15 mM PBS buffer (pH 7.0) was incubated at 0 °C and 45 °C, separately. After 15 minutes, the reaction was quenched by adding 2 mL of DNS reagent and boiled for 5 min. The absorbance was measured at 540 nm. One unit of hydrolytic activity was defined as the quantity of enzyme that released reducing sugar equivalent to one μmol of glucose per minute under the assay conditions. Relative activity was quantified as the percentage ratio of enzymatic activity observed at 0 °C to that at 45 °C (the optimal temperature of psA). Three biologically independent replicates were used to calculate mean and standard deviation.

## RESOURCE AVAILABILITY

### Lead contact

Requests for further information and resources should be directed to the lead contact, Zhongyue J. Yang (zhongyue.yang@vanderbilt.edu)

### Materials availability

Example dialog between user and MutexaGPT for setting up the cavity study; Example dialog between user and MutexaGPT for setting up the cold-adaption study; The design of each individual LLM-agent; The evaluation system of LLM-agents; An example output of ResultExplainer; Topology-based selection of psA linkers; System prompts of all LLM-agents involved in this study; Evaluation results for LLM-agents in MutexaGPT; Linker sequences used in cold-adaption engineering and their calculated Domain Separation Index (DSI); The DSI distribution of the *Pseudomonas saccharophila* amylase wild type and its ten linker variants; Amylase activity of psA wild-type, psAt5, and psAt6 under 0 °C and 45 °C; Full sequences of psA variants; Parameters used for structure prediction using ColabFold server. (Supporting Information PDF)

### Data and code availability

The MutexaGPT webapp is available at https://enzyhtp.app.vanderbilt.edu/. To access the webapp, one can use the account name: mutexatest@testemail.com along with the password: mutexatest111. All test cases can be found under this account. The source code of MutexaGPT and the evaluation system of LLM-agents in MutexaGPT is available from https://github.com/ChemBioHTP/EnzyHTP-GPT/. EnzyHTP is available from https://github.com/ChemBioHTP/EnzyHTP. AMBER is available from http://ambermd.org/. The original input file for all MD involved is available from Zenodo (https://doi.org/10.5281/zenodo.17905121).

## Supplementary Material

Supplementary Files

This is a list of supplementary files associated with this preprint. Click to download.

- SI.pdf
